# Therapeutic patterns and outcomes in older patients (aged ≥65 years) with stage II-IVB Nasopharyngeal Carcinoma: an investigational study from SEER database

**DOI:** 10.7150/jca.46201

**Published:** 2020-07-09

**Authors:** Yongfeng Piao, Chuner Jiang, Fengqin Yan, Zhimin Ye, Zhenfu Fu, Haitao Jiang, Yangming Jiang, Yuezhen Wang, Fangzheng Wang

**Affiliations:** 1 Department of Radiation Oncology, Cancer Hospital of the University of Chinese Academy of Sciences, Zhejiang Hangzhou 310022, People's Republic of China.; 2 Institute of Cancer and Basic Medicine (ICBM), Chinese Academy of Sciences, Zhejiang Hangzhou 310022, People's Republic of China.; 3 Department of Radiation Oncology, Zhejiang Cancer Hospital, Zhejiang Hangzhou, 310022, People's Republic of China.; 4 Key Laboratory of Radiation Oncology of Zhejiang Province, Zhejiang Hangzhou, 310022, People's Republic of China.; 5 Department of Breast Tumor Surgery, Cancer Hospital of the University of Chinese Academy of Sciences, Zhejiang Hangzhou 310022, People's Republic of China.; 6 Department of Breast Tumor Surgery, Zhejiang Cancer Hospital, Zhejiang Hangzhou, 310022, People's Republic of China.; 7 Department of Radiology, Cancer Hospital of the University of Chinese Academy of Sciences, Zhejiang Hangzhou 310022, People's Republic of China.; 8 Department of Radiology, Zhejiang Cancer Hospital, Zhejiang Hangzhou, 310022, People's Republic of China.; 9 Department of Didital Earth, Institute of Remote Sensing and Didital Earth, CAS, Beijing, 100101, People's Republic of China.

**Keywords:** nasopharyngeal carcinoma, older, therapeutic pattern, survival, prognosis

## Abstract

**Purpose:** Currently, the optimal treatment regimens for older nasopharyngeal carcinoma (NPC) patients remained unclear. The aim of this retrospective study is to investigate therapeutic patterns and survival outcomes for a cohort of older NPC patients receiving radiation therapy (RT) with or without chemotherapy (CT).

**Methods:** The clinical data of 529 patients with aged ≥65 years and NPC, who were identified within the Surveillance, Epidemiology, and End Results (SEER) registry (years 2004-2015), were collected and retrospectively reviewed. Among these patients, 74 patients treated with RT alone and 455 cases were administrated for RT plus CT. Kaplan-Meier analysis was used to evaluate overall survival (OS) and cancer-specific survival (CSS). The differences in OS and CSS were compared using Log-rank test.

**Results:** The estimated OS and CSS rates at 5 years were 48.9% and 59.6%, respectively. Univariate analysis indicated that age, histology, T stage, and clinical stage were independent prognosticators of OS and CSS, while treatment option was only associated with OS. Multivariate analysis demonstrated that age, T stage, histology, and therapeutic strategy were correlated with OS, while age, T stage and histology were independent prognostic factors of CSS. Subgroup analyses showed that the combination of RT and CT yielded better OS and CSS in patients with stage T3 or N2 or III.

**Conclusion:** Among these NPC patients with aged ≥65 years reported in the SEER database, treatment with RT plus CT provided longer OS than those treated with radiation therapy alone. Moreover, the combination of RT and CT obtained favorable OS and CSS in NPC patient stage T3 or N2 or III.

## Introduction

Nasopharyngeal cancer (NPC) is a unique malignancy of the head and neck, with acute incidence of 15-50 cases per 100,000 annually in endemic areas such as south-east Asia, northern Africa and middle Europe 1. The GLOBOCAN data 2 in 2018 reported that there are 129097 patients with newly diagnosed NPC around the world. Of these patients, the rate of Chinese patients accounted for 47.7%. The proportions of NPC vary with age, ethnicity and geographical origin in the endemic area 1. Moreover, the peak age was between 40 and 60 years and subsequently reduced in the elderly 3. Furthermore, the older patients with NPC are improving gradually in the future due to life expectancy and the improvement in the number of older population. However, there was no evidence of the standard therapy for the older patients, since patients aged ≥ 65 years were either excluded from, or accounted for few, clinical trials 4-6. Thence, appropriate therapeutic strategy for the older NPC patients remained unclear.

The latest NCCN guideline in 2020 7 recommended that radiation therapy (RT) alone is used to treat for stage I NPC and concurrent chemoradiotherapy (CCRT) with induction chemotherapy (IC) or adjuvant chemotherapy (AC) is recommended as the first line treatment for locoregionally advanced NPC. The therapeutic schemes recommended by NCCN guideline were mainly used for the younger NPC patients. Thus, the treatment regimens administrated for the newly diagnosed NPC older patients referred to those for the younger patients. The two previous retrospective studies indicated that conventional RT combined with chemotherapy (CT) provided appropriate survival outcomes and manageable adverse events in older NPC patients with favorable performance status 8,9. However, the proportion of the older patients who treated with RT plus CT in these was less than 40%. In addition, an investigational study conducted by Vivek et al indicated that CCRT, compared to RT alone, provided better survival outcomes in aged ≥ 70 years NPC patients 10. While a pair-matched study performed by Mi et al showed that compared with intensity-modulate radiotherapy (IMRT) alone, adding CT to IMRT provided comparable survival and more grade 3 toxicities 11. Given these situations, most oncologists usually recommended RT alone for treating older NPC patients 12,13. Thence, the present study analyzed the therapeutic patterns and survival in aged ≥65 year-old patients with NPC.

## Materials and Methods

### Database and patients' selection

All histology-proven patients with NPC were identified in the Surveillance, Epidemiology, and End Results (SEER) database of the National Cancer Institute in the American. The SEER 18 database 14 was obtained in the SEER program with SEER*Stat software, version 8.3.6 (www.seer.cancer.gov/seerstat). We selected the patients using the criteria as following: the patients were selected if age at diagnoses older than 65 years and diagnosed with NPC as the first malignancy from 2004 to 2015. And we used the American Joint Committee on Cancer 6^th^ or 7^th^ edition staging to define the patients' stage. Stage UNK or distant metastases (M1) were exclusions. The therapeutic scheme for the NPC patients with aged ≥65 years were either RT or RT plus CT. Subjects received no treatment were excluded.

**Figure 1:**
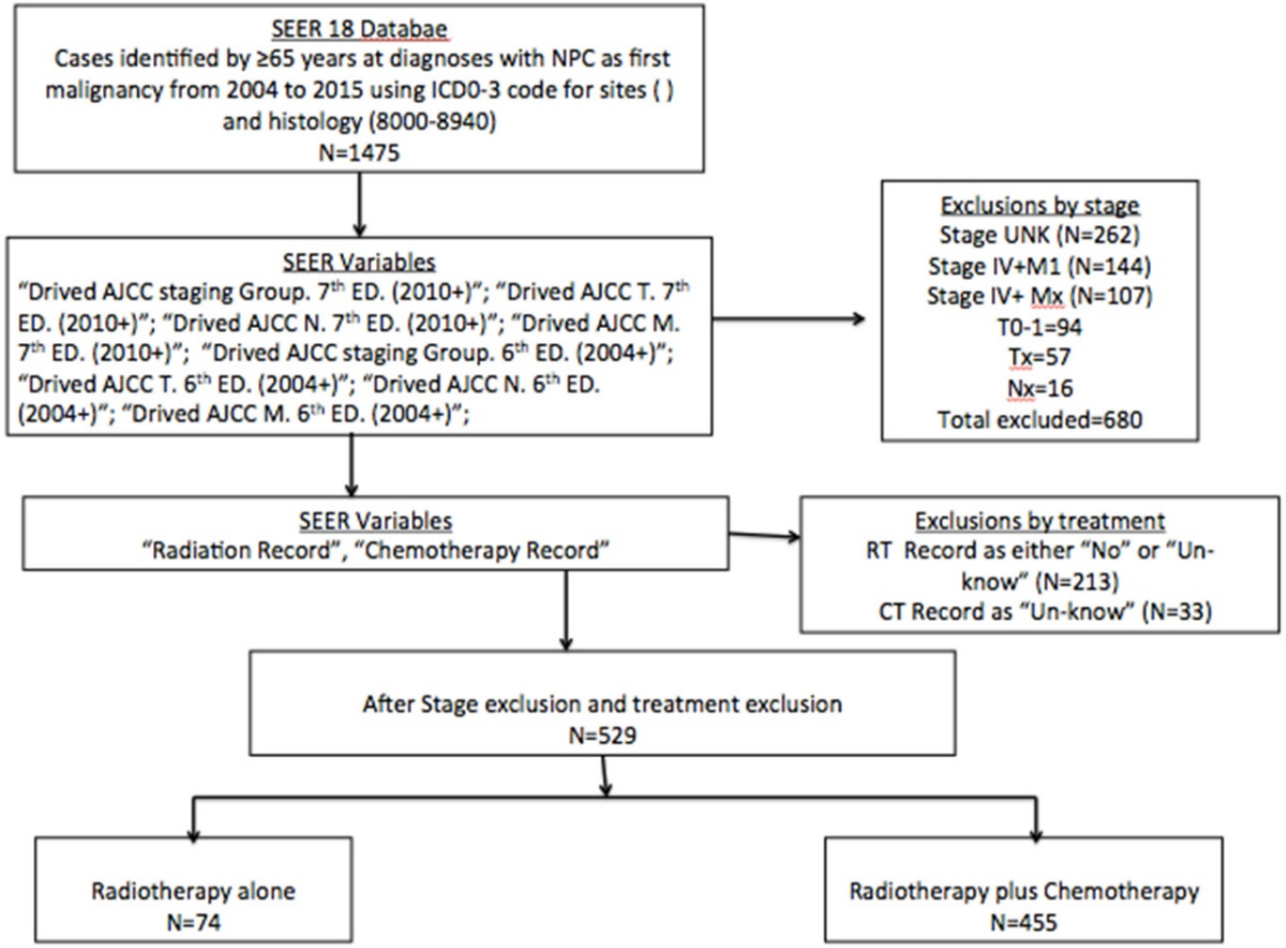
Flowchart of selective patients with aged ≥70 years and nasopharyngeal carcinoma diagnosed from 2004 to 2015 in the SEER registry based on therapy option received. Abbreviations: NPC: nasopharyngeal carcinoma; ICD-03: International Classification of Diseases for Oncology, 3^rd^ Edition; AJCC: American Joint Committee on Cancer; SEER: Surveillance, Epidemiology, and End Results.

The flowchart of selective patients is shown in Figure 1. A total of 1475 histology-confirmed NPC patients were registered in SEER database. A total of 529 NPC patients with aged ≥65 years received RT with or without CT were respectively reviewed. The study was exempt from our Institution Review Board because the information of all patients was publicly available. The information collected prospectively included patient demographics, histology, stage, therapeutic strategy, overall survival (OS) and cancer-specific survival (CSS).

### Statistical analyses

The trends in treatment option, 5-year OS and CSS was carried out by using SEER*Stat. All other data was analyzed by using IBM SPSS Statistical software, version 25.0. We used vital status and follow-up time from diagnosis date to calculated OS. And we used the cancer-specific death classification to compute CSS. The Kaplan-Meier method was used to generate survival curves and log-rank tests were used compare the curves of different variables. We used a Cox regression model to conduct the multivariate in order to identify significant prognosticators. And we calculated hazard ratios (HRs) and 95% confidence intervals (CIs) for each prognosticator. If a *P* value < 0.05, the differences were considered as statistically significant.

## Results

### Patients' characteristics

There were 529 patients with a mediate age of 71 years (range 65-88 years). Of these patients, the rate of male was 64.5%, non-Hispanic White was 51.3%, and stage III-IV was 66.5%. 74 patients received RT alone and 455 patients treated with RT plus CT. Table 1 summarized the cohort characteristics.

### Survival analyses

**Figure 2:**
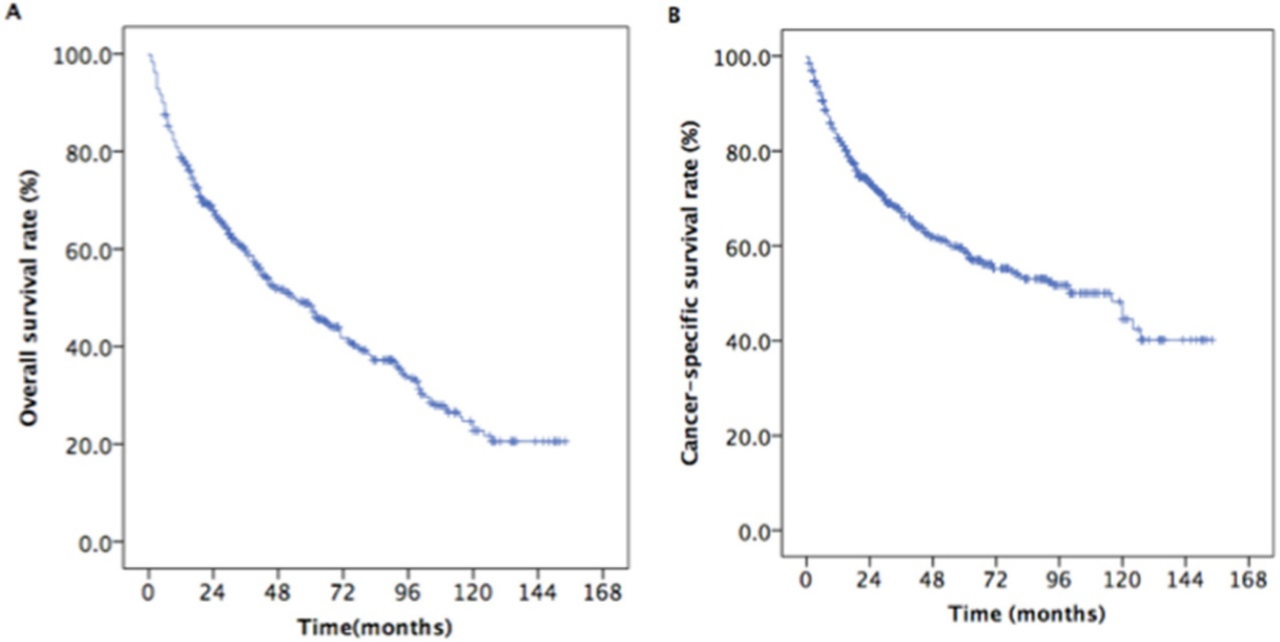
Kaplan-Meier estimates of the survival in newly diagnosed nasopharyngeal carcinoma patients with aged ≥ 65years. (A) Overall survival; (B) Cancer-specific survival.

**Figure 3:**
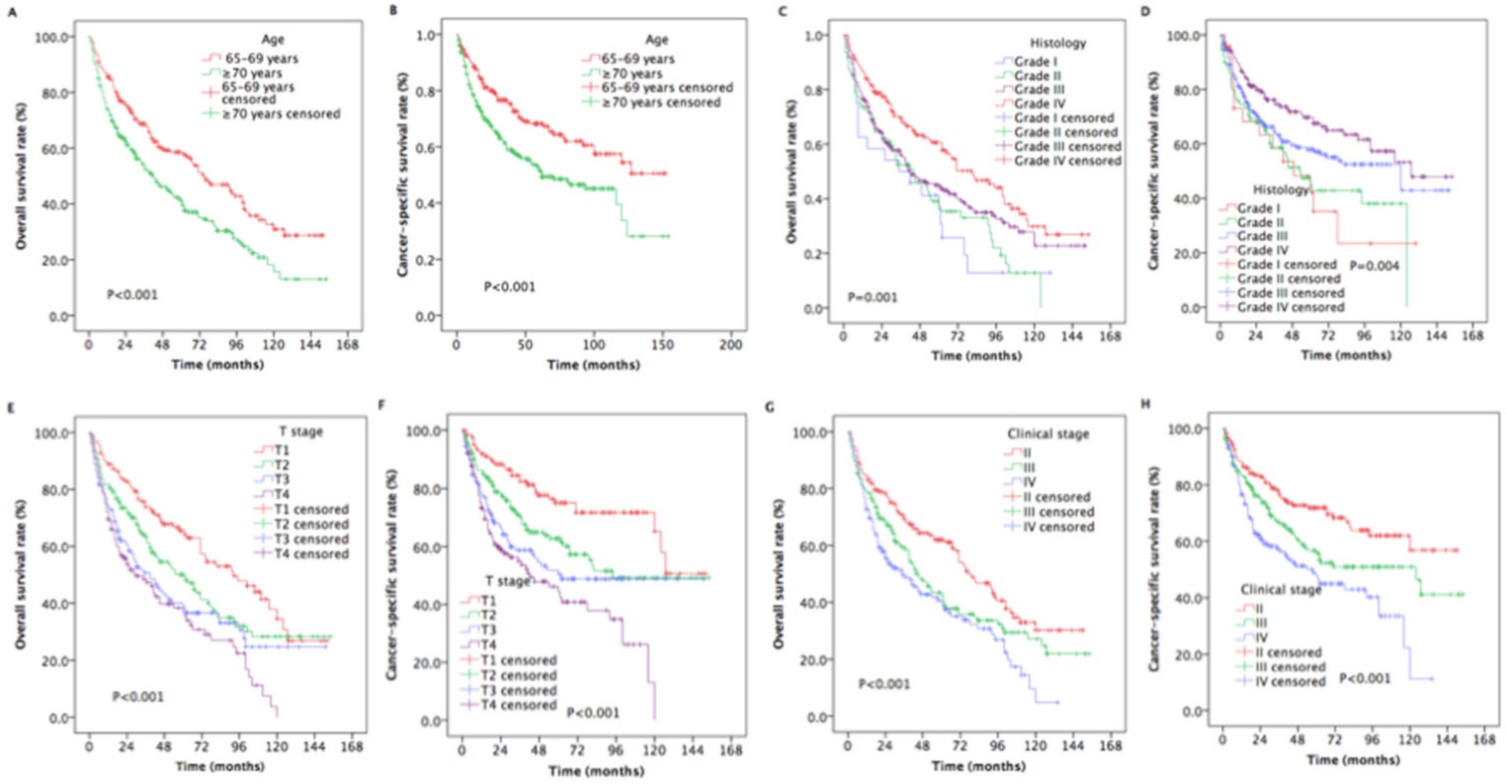
Kaplan-Meier estimates of the survival in newly diagnosed nasopharyngeal carcinoma patients with aged ≥ 65 years for different variables. (A) Overall survival for age; (B) Cancer-specific survival for age; (C) Overall survival for histology; (D) Cancer-specific survival for histology; (E) Overall survival for T stage; (F) Cancer-specific survival for T stage; (G) Overall survival for clinical stage; (H) Cancer-specific survival for clinical stage.

**Figure 4:**
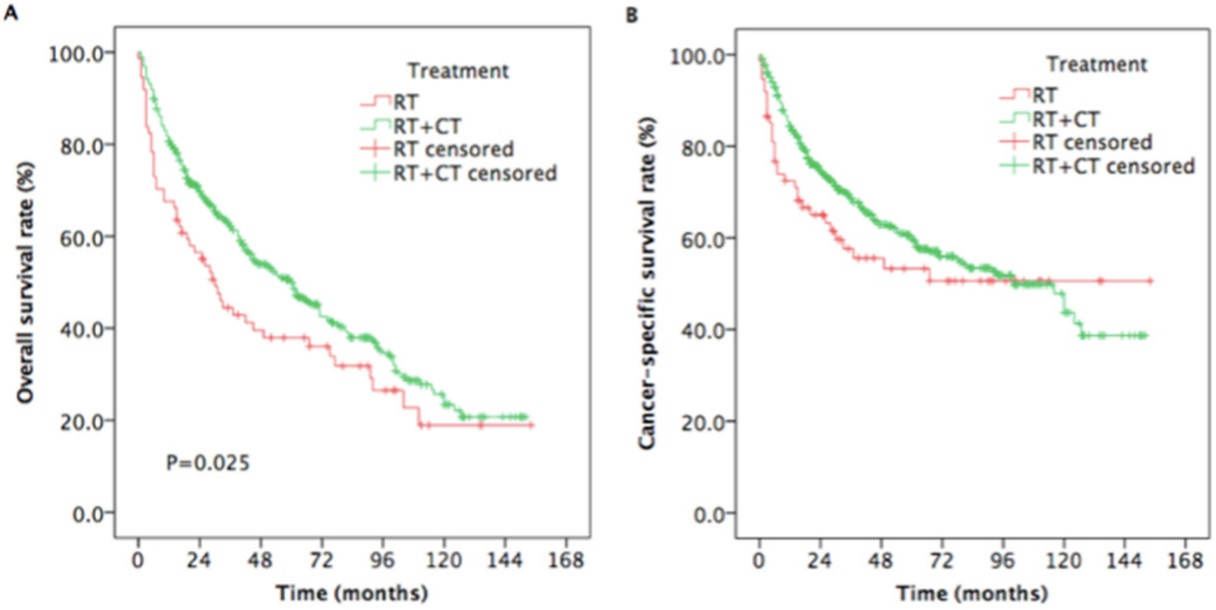
Kaplan-Meier estimates of the survival in newly diagnosed nasopharyngeal carcinoma, aged ≥ 65 years patients treated with RT and RT plus CT. (A) Overall survival; (B) Cancer-specific survival. Abbreviations: RT: radiotherapy; CT: chemotherapy.

The median survival time was 33 months (range, 3 to 154 months). The estimated OS and CSS rates at 5 years were 48.9% and 59.6%, respectively (Figure 2). The estimated rates OS at 5 years were 58.5% and 42.0% for patients with aged 65-70 years and ≥ 70 years, respectively (*P* < 0.001, Figure 3A). The estimated 5-year CSS rates were 68.3% and 53.1% for patients with aged 65-69 years and ≥ 70 years, respectively (*P* < 0.001, Figure 3B). The estimated rates OS at 5 years were 36.1%, 37.3%, 45.0%, and 60.7% for patients with grade I, II, III, and IV, respectively (*P* =0.001, Figure 4C). The estimated 5-year CSS rates were 42.2%, 45.1%, 58.0%, and 69.7% for patients with grade I, II, III, and IV, respectively (*P* < 0.001, Figure 4D). The estimated 5-year OS rates were 65.5%, 50.1%, 38.29%, and 36.9% for patients with stage T1, T2, T3and T4, respectively (*P* < 0.001, Figure 3E). The estimated CSS rates at 5 years were 75.0%, 62.7%, 50.3%, and 44.4% for patients with stage T1, T2, T3and T4, respectively (*P* < 0.001, Figure 3F). The estimated rates OS at 5 years were 62.0%, 42.6%, and 39.6% for patients with stage II, III, and IV, respectively (*P* < 0.001, Figure 4G). The estimated 5-year CSS rates were 71.8%, 55.7%, and 47.5% for patients with stage II, III, and IV, respectively (*P* < 0.001, Figure 4H).

The estimated 5-year OS rates were 38.0% and 50.7% for patients treated with RT and RT plus CT, respectively (*P* =0.025, Figure 4A). The estimated 5-year CSS rates were 53.3%, and 60.6% for patients treated with RT and RT plus CT, respectively (*P* = 0.176, Figure 4B).

### Identification of prognostictors

Several potential prognostictors such as age, gender, histology, race, T stage, N stage, clinical stage, and therapeutic regimen. We used univariable Cox regression to evaluate these prognostic factors, and the results showed that age, histology, T stage, and clinical stage were significant prognosticators for OS and CSS, while therapeutic strategy was only associated with OS (Table 2).

**Figure 5:**
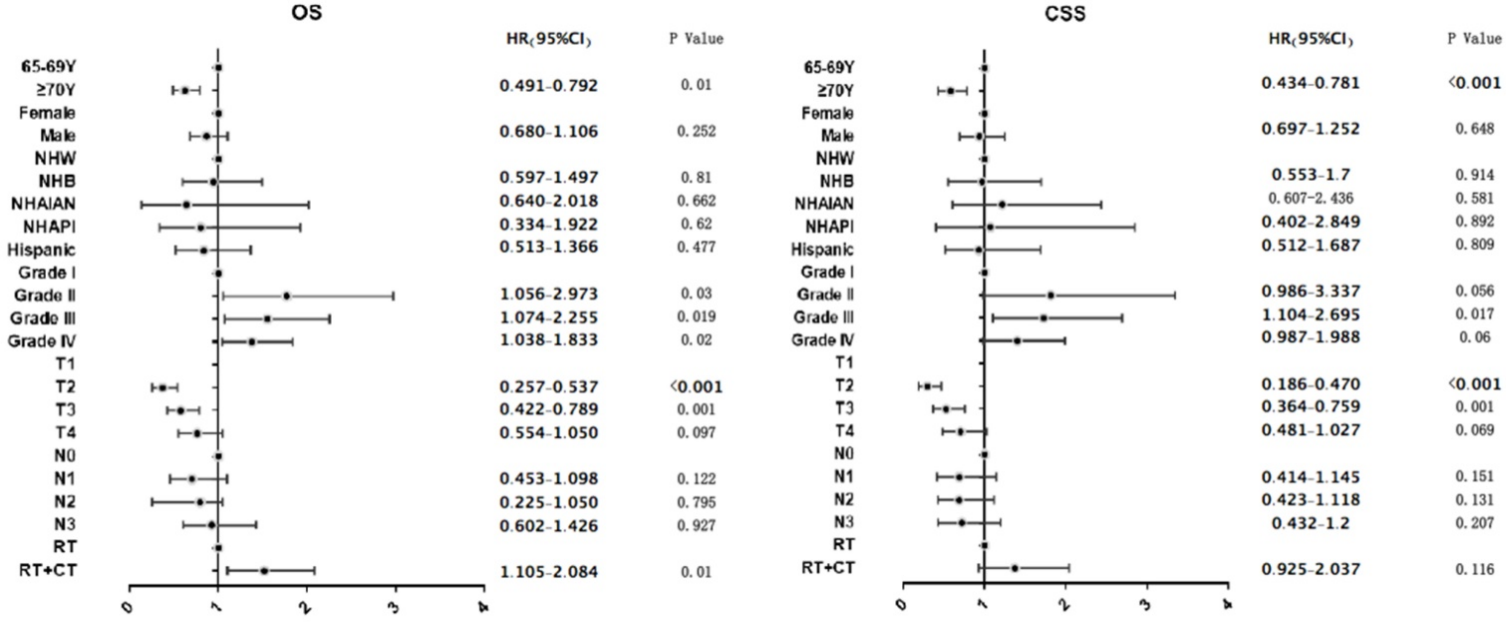
Weighted multivariable Cox proportional hazards regression analyses are shown for overall survival and cancer-specific survival; NHAIAN Non-Hispanic American Indian/Alaska Native; NHAPI Non-Hispanic Asia or Pacific Islander; NHB Non-Hispanic Black; NHW Non-Hispanic White; RT radiotherapy; CT chemotherapy; NPC nasopharyngeal carcinoma; OS overall survival; CSS Cancer-specific survival; * The 6^th^ or 7^th^ AJCC/UICC staging system.

Multvariable Cox regression demonstrated that treatment with RT + CT was associated with longer OS compared to treatment with RT alone (HR=1.518, 95% CI: 1.105-2.084, P=0.01; Figure 5A), while RT plus CT, compared to RT alone, did not provide the advantages of CSS (HR=1.372, 95% CI: 0.925-2.037, P=0.116; Figure 5B). Moreover, aged ≥70 years compared to aged 65-69 years was associated with poorer OS and CSS (OS HR=0.624, 95% CI: 0.491-0.792, P<0.001; CSS HR=0.582, 95% CI: 0.434-0.781, P<0.001; Figure 5).

### Subgroup analysis

In order to identify which newly diagnosed NPC patients with aged ≥65 years would benefit from the combination of RT and CT, we performed a subgroup analysis. RT combined with CT provided longer OS (P=0.003) and CSS (P=0.018) in patients with stage T3 (Table 3). Furthermore, the patients with stage N2 or III could gain more advantages of OS and CSS from RT plus CT. Interestingly, the male patients had more favorable OS (P=0.037) from RT plus CT.

## Discussion

To our best knowledge, the standard treatment regimens for the older patients remained unproven since the patients aged ≥65 years were excluded from any clinical trial. In the present study of newly diagnosed NPC patients with over 65 years, we carefully identified the appropriate patients from the 18 SEER database and investigated the therapeutic patterns and survival outcomes. These findings indicated that the combination of RT and CT was associated with significantly longer OS.

On our univariate and multivariate analysis, RT plus CT, compared to RT alone, was associated with a significant improvement in OS, while did not provide longer CSS. Moreover, aged ≥70 years compared to aged 65-69 years was associated with poorer OS and CSS. Subgroup analysis showed that RT combined with CT provided longer OS and CSS in patients with stage T3. Furthermore, the patients with stage N2 or III could gain more advantages of OS and CSS from RT plus CT. Interestingly, the male patients also had more favorable OS from RT plus CT.

Because of extension of lifespans and demographic changes in the age, more and more elderly patients had been diagnosed with NPC 8,15. The older NPC patients, in contrast to younger cases, had a significantly higher risk of death and cancer progression 16. Therefore, it is very important for elderly NPC patients to select the appropriate therapeutic schedule. In the era of conventional RT, the combination of RT and CT provided survival advantages for older NPC patients 17-20. Moreover, the aged >65 years NPC patients received insufficient cycles and doses of CT due to the poor performance status 8. Vivek et al indicate that compared with RT alone, CCRT provided favorable survival outcomes in aged ≥ 70 years NPC patients 10, while Mi et al showed that IMRT alone, compared to IMRT plus CT, provided less grade 3 side events and comparable survival with 3-year of OS (72.1% vs. 72.5%, P=0.735), PFS (65.9% vs. 70.1%, P=0.735), DMFS (76.4% vs. 71.6%, P=0.735) and LRRFS (90.8% vs. 98.0%, P=0.735) 11.

Furthermore, the unfavorable factors including poor performance status, comorbidities, reduced organ function and decreased social support affected the tolerance of CCRT in the older patients. More grade 3 and 4 CT-related side events were correlated with the cancer patients with the comorbidities 21. Goto et al indicated that the factors such as comorbidities, stage and administration, were associated with poor prognoses in older population 22. Several retrospectively studies reported that therapy-related complications were not increased in the elderly patients 23,24, while other studies reported that the proportions of adverse events were improved with increasing patients' age 25,26. In addition, the older patients experienced comorbidities and metabolic changes 16, and more treatment-related complications occurred in the older patients due to these disease 21,27. Vercelli et al revealed that improving complications were closely related with poor prognosis in older patients 28. To illustrate the role of performance status on survival, Muller et al showed that the patients with ECOG performance status of 2-3 resulted in poor OS and PFS 29. Considering these factors, appropriate therapeutic schedules were recommended for treating the older patients.

In order to identify which newly diagnosed NPC patients with aged ≥65 years would benefit from the combination of RT and CT, we performed a subgroup analysis. Treatment with RT and CT provided longer OS and CSS in patients with stage T3. Furthermore, the patients with stage N2 or III could obtain more benefits of OS and CSS from RT plus CT. Interestingly, the male patients had more favorable OS from RT plus CT. According to these results, appropriated therapeutic strategy was used for each older NPC patients.

Although SEER database provides a public available data to investigate this clinical problem, several limitations were observed in this study. Firstly, treatment information including RT dosing, CT regimen, delays in CT and therapy dates was not registered into SEER database, and we did not analyze the role of these factors. Secondly, therapy-related complications were not evaluated in this study due to lack of the information about RT- and CT-related side events in the SEER database. In addition, SEER records did not include the information about locoregional relapse, distant metastasis, so we failed to assess LRRFS and DMFS.

Although there are some limitations in the present study, it indicated that RT combined with CT provided the benefits of OS and CSS in the NPC patients with aged ≥ 65 years. Moreover, subgroup analysis showed that adding CT to RT provided longer OS and CSS in patients with stage T3 or N2 or III. Further directions include appropriate CT regimen used to treat older NPC patients, target treatment used in the treatment of older NPC patients, screening out appropriate older patients benefit from the combined treatment. Thence, the prospective phase III trials needed to validate these gains.

## Conclusion

The current study indicated that compared with RT alone, the combination of RT and CT provided longer OS and CSS in newly diagnosed NPC patients with aged ≥65 years from the 18 SEER database. Moreover, the patients with stage T3 or N2 or III may be administrated with RT plus CT. It needs to conduct further multicenter prospective clinical trials to verify the ultimate benefits.

### Author contributions

**Conception and design:** Fangzheng Wang, Haitao Jiang, Zhenfu Fu, Yangming Jiang.

**Acquisition of data:** Chuner Jiang, Fengqin Yan, Zhimin Ye, Yongfeng Piao.

**Data analysis and interpretation:** Fangzheng Wang, Yangming Jiang.

**Drafting the article and revising it critically for important intellectual content:** Yongfeng Piao, Fangzheng Wang, Yuezhen Wang, Yangming Jiang.

**Final approval of manuscript:** All authors.

### Funding

This study was supported by grants from the Medical and Health Science and Technology Program of Zhejiang Province (No.2020KY084, No.2019KY041, No. 2013KYB033, No. 2009B026, No. 2006A016, No. 2005B012, No. 2004B014), National Natural Science Foundation of China (No. 81502647).

## Figures and Tables

**Table 1:** Basic characteristics of selected aged ≥65 years NPC patients with stage II-IVB from 2004 to 2015 in the SEER Database

Characteristics	N (%)
**Age**	
Mediate (year)	71
Rang (year)	65-88
< 70 years	222 (42.0)
≥ 70 years	307 (58.0)
**Gender**	
Male	341 (64.5)
Female	188 (35.5)
**Race**	
Hispanic	36 (6.8)
NHAIAN	15 (2.8)
NHAPI	165 (31.2)
NHB	42 (7.9)
NHW	271 (51.3)
**Grade**	
Grade I	24 (4.5)
Grade II	80 (15.1)
Grade III	253 (47.8)
Grade IV	172 (32.6)
T stage*	
T1	124 (23.4)
T2	164 (31.0)
T3	126 (23.8)
T4	115 (21.8)
N stage*	
N0	139 (26.3)
N1	208 (39.3)
N2	130 (24.6)
N3	52 (9.8)
Clinical stage*	
II	177 (33.5)
III	190 (35.9)
IV	162 (30.6)
**Treatment option**	
RT	74 (14.0)
RT+CT	455 (86.0)

Abbreviations: NHAIAN Non-Hispanic American Indian/Alaska Native; NHAPI Non-Hispanic Asia or Pacific Islander; NHB Non-Hispanic Black; NHW Non-Hispanic White; RT radiotherapy; RT radiotherapy; CT chemotherapy; * The 6^th^ or 7^th^ AJCC/UICC staging system.

**Table 2:** Results of univariable analysis of both OS and CSS in selected newly diagnosed NPC patients with ≥ 65 years in the 18 SEER database

Characteristics	5-year OS	P	5-year CSS	P
**Age**				
65-79 years	58.5	<0.001	68.3	<0.001
≥ 70 years	42.0		53.1	
**Sex**				
Female	50.2	0.449	60.1	0.953
Male	47.3		58.1	
**Histology**				
Grade I	36.1	0.001	42.2	0.004
Grade II	37.3		45.1	
Grade III	45.0		58.0	
Grade IV	60.7		69.7	
**Race**				
NHW	47.4	0.606	57.2	0.845
NHB	33.8		47.7	
NHAIAN	66.7		66.7	
NHAPI	50.6		62.7	
Hispanic	53.2		62.6	
T stage *				
T1	65.5	<0.001	75.0	<0.001
T2	50.1		62.7	
T3	38.9		50.3	
T4	36.9		44.4	
N stage *				
N0	44.0	0.177	51.4	0.054
N1	56.1		65.9	
N2	42.9		57.8	
N3	42.9		44.4	
Clinical stage *				
II	62.0	<0.001	71.8	<0.001
III	42.6		55.7	
IV	39.6		47.5	
**Therapy strategy**				
RT	38.0	0.025	53.3	0.176
RT+CT	50.7		60.6	

Abbreviations: NHAIAN Non-Hispanic American Indian/Alaska Native; NHAPI Non-Hispanic Asia or Pacific Islander; NHB Non-Hispanic Black; NHW Non-Hispanic White; RT radiotherapy; CT chemotherapy; NPC nasopharyngeal carcinoma; OS overall survival; CSS Cancer-specific survival; * The 6^th^ or 7^th^ AJCC/UICC staging system.

**Table 3:** Subgroup analysis of both OS and CSS in newly diagnosed NPC patients with age ≥ 65 years in the 18 SEER database treated with RT, CT or RT plus CT

Characteristics	CSS		P	OS		P
	RT	RT+CT		RT	RT+CT	
**Age**						
65-69 years	66.1	68.5	0.838	50.5	59.6	0.307
≥ 70 years	46.3	54.5	0.231	31.8	43.9	0.093
**Gender**						
Female	49.8	59.2	0.345	30.7	49.6	0.037
Male	56.1	60.8	0.328	46.7	50.8	0.268
**Histology**						
Grade I	25	46.7	0.082	25	38.6	0.813
Grade II	49.1	44.1	0.711	36.4	36.7	0.411
Grade III	55.8	58.6	0.186	33.9	47.4	0.015
Grade IV	63	70.4	0.963	56.0	61.2	0.566
T stage *						
T1	87.8	73.5	0.381	52.3	67.6	0.027
T2	60	63.3	0.830	46.9	50.8	0.969
T3	27.3	54.3	0.003	19.4	42.3	0.018
T4	22.9	46.0	0.065	20	38.2	0.062
N stage *						
N0	51.0	51.7	0.627	40.9	44.8	0.552
N1	68.5	65.6	0.812	50.2	56.8	0.182
N2	15.2	74.7	0.020	9.1	46.4	0.012
N3	64.3	51.1	0.553	26.8	44.4	0.918
Clinical stage *						
II	86.1	69.2	0.169	57.7	61.3	0.987
III	25.4	60.9	<0.001	15.6	47.7	<0.001
IV	42.0	48.3	0.342	25.7	41.2	0.123

Abbreviations: RT radiotherapy; CT chemotherapy; NPC nasopharyngeal carcinoma; SEER Surveillance, Epidemiology, and End Results; OS overall survival; CSS cancer-specific survival; * The 6^th^ or 7^th^ AJCC/UICC staging system.
